# The clinical, serological and myopathological features of a cohort of Chinese patients with inclusion body myositis: a single center analysis

**DOI:** 10.3389/fimmu.2026.1782053

**Published:** 2026-03-05

**Authors:** Hongyan Qiu, Shouzheng Yang, Xuejun Guo, Yinglin Leng, Yawen Zhao, Meng Yu, Yiming Zheng, Lingchao Meng, He Lv, Jianwen Deng, Wei Zhang, Zhaoxia Wang, Yun Yuan, Qiang Gang

**Affiliations:** 1 Department of Neurology, Peking University First Hospital, Beijing, China; 2 Rare Diseases Medical Center, Peking University First Hospital, Beijing, China; 3 Department of Neurology, Peking University Shenzhen Hospital, Shenzhen, China; 4 Department of Neurology, Beijing Jishuitan Hospital, Capital Medical University, Beijing, China

**Keywords:** anti-cN1A antibody, complement deposition, dysphagia, IBM, inclusion body myositis

## Abstract

**Background:**

This study aimed to investigate the clinical, serological and myopathological features of Chinese patients with inclusion body myositis (IBM).

**Methods:**

This study retrospectively recruited patients diagnosed with IBM according to the 2024 European Neuromuscular Center (ENMC) criteria at Peking University First Hospital between 2004 and 2024. Clinical features, pathological changes and laboratory data were collected. Subgroups were analyzed by sex, dysphagia, anti-cN1A antibody status, and complement deposition.

**Results:**

Forty-three IBM patients (25 males) were included in this cohort. The mean age at onset was 54.9 ± 9.6 years old. All the patients developed weakness in hip flexion, and 81.4% of them with weakness in both knee extension and finger flexion. Dysphagia was reported in 17 patients (39.5%). Endomysial inflammation was observed in all the patients, and 79.1% with rimmed vacuoles, and 76.9% with mitochondrial abnormalities. Female patients more frequently developed dysphagia (P = 0.005) and neck flexion weakness (P < 0.001). Anti-cN1A antibody was positive in 20 patients (66.7%). Seropositive cases were associated with a later age of onset (P = 0.034). Complement deposition was observed in 77.4% of patients and was associated with more severe muscle weakness.

**Conclusion:**

This cohort of Chinese IBM patients suggested an earlier age at onset than previously reported. Hip flexors were most commonly affected. Female patients showed a higher frequency of dysphagia and neck flexor weakness. Our study reported a high frequency of complement deposition in muscle tissue. Complement deposition was associated with disease severity, suggesting a potential role of complement in the pathophysiology of IBM.

## Introduction

Inclusion body myositis (IBM) is the most common inflammatory myopathy in Caucasian patients aged over 50 years, characterized by slowly progressive muscle weakness, predominantly in the flexor digitorum profundus and quadriceps femoris (1). The diagnosis of IBM relies on both clinical and pathological features. The 2024 revised European Neuromuscular Center (ENMC) diagnostic criteria specifies endomysial lymphocytes surrounding non-necrotic muscle fibers (with or without invasion) as mandatory myopathological feature, and rimmed vacuoles and/or cytoplasmic protein aggregates and mitochondrial abnormalities as supportive features (2). Due to the heterogeneity of clinical and pathological findings, IBM is frequently misdiagnosed at initial clinic visit. The 2024 ENMC criteria for the first time incorporated atypical presentations, including early-onset disease, axial weakness, isolated dysphagia, foot drop, facial diplegia and proximal limb weakness (2). The etiology of IBM remains elusive (3, 4), and it is refractory to conventional immunotherapy, often leading to significant functional decline in daily activities among elderly patients (5).

Over the past decade, the serum anti-cytosolic 5’-nucleotidase 1A (cN1A) antibody has emerged as a potential biomarker for IBM (6). However, its reported sensitivity varies ranging from 37% in European cohorts to approximately 60% in North American studies and up to 80% in Japanese patients (7–9). In addition, anti-cN1A antibodies are not specific to IBM and can also be detected in other idiopathic inflammatory myopathies, Sjögren’s syndrome, and systemic lupus erythematosus (8, 10). It has remained under debate whether the clinicopathological features were different between seropositive and seronegative IBM patients. Some studies reported that seropositive patients present with more severe motor or bulbar involvement (11, 12). Some studies described fewer rimmed vacuoles or a higher burden of cytochrome c oxidase (COX)-deficient fibers in seropositive cases (7, 8), whilst others found no significant clinicopathological differences between seropositive and seronegative cases (13–15).

IBM has been rarely reported in China, mostly described in case reports and small cohorts (16, 17). In a study of 28 Chinese patients, quadriceps were the most commonly affected, while conspicuous muscle atrophy in quadriceps was observed in only 17.9% (16). In addition, the anti-cN1A antibody has never been systematically studied in Chinese patients. This highlights the need for comprehensive clinical and pathological profiling of IBM in Chinese population.

Therefore, this study aimed to characterize the clinical, serological and myopathological features of Chinese patients with IBM.

## Materials and methods

### Patients

Medical records and muscle histopathology were retrospectively reviewed for the patients who were diagnosed as “IBM” or “possible IBM” at Peking University First Hospital (PKUFH) between 2004 and 2024. The patients fulfilling the 2024 ENMC criteria for IBM were recruited to this study (2). Clinical information included the following items: age at onset, sex, disease duration, age at biopsy, diagnostic interval, past medical history, symptoms at onset, muscle weakness distribution, comorbidities, serum creatine kinase (CK) level, and serum anti-cN1A antibody status. In this study, age at onset referred to the date when symptoms of IBM were reported by the patient. Disease duration was defined as the time from disease onset to the time of assessment. Diagnostic interval referred to the time from onset to the diagnosis.

Manual muscle strength was recorded in 13 muscle groups using the Medical Research Council (MRC) scale, including neck flexors, shoulder abductors, elbow flexors and extensors, wrist flexors and extensors, finger flexors and extensors, hip flexors, knee flexors and extensors, ankle dorsiflexors and plantar flexors (18, 19). MRC grades were further converted to a 10-point Kendall scale for analysis according to the previous studies (19, 20). The conversion criteria were provided in **Supplementary Table 1**. Muscle strength of each muscle group was calculated as the average of both sides of the limbs. Muscle strength sum score (SSS) was the total score of all the muscle groups, ranging from 0 (complete paralysis of the included muscles) to 130 (normal). IBM functional rating scale (IBMFRS) scores were assessed during the physical examination. All clinical assessments were performed by two neuromuscular specialists at PKUFH.

Serum anti-cN1A antibody was measured using a commercially available enzyme-linked immunosorbent assay (ELISA) kit (EUROIMMUN, Lübeck, Germany), or commercial cell-based assay (CBA) method (KingMed Diagnostics, Guangzhou, China). The choice of method for antibody testing depended on which method was available at the time of evaluation.

### Muscle biopsy

The muscle biopsies from our center were reviewed and analyzed by two neuropathologists. Histochemistry stainings included hematoxylin and eosin (H&E), modified Gömöri trichrome (mGT) and cytochrome c oxidase/succinate dehydrogenase (COX/SDH) staining. Immunohistochemistry stainings were performed using antibodies against the following antigens: major histocompatibility complex class I (MHC-I), CD3, CD4, CD8, CD20, CD68, C5b-9, p62 and TDP-43. The available electron microscopy (EM) slides were also thoroughly reviewed. Histopathological features were recorded, including perimysial inflammation, endomysial inflammation, endomysial fibrosis, inflammatory invasion or infiltration of non-necrotic muscle fibers, rimmed vacuoles (RVs), MHC-I, mitochondrial abnormalities, complement deposition and p62 protein aggregates on light microscopy, and tubulofilamentous inclusions on the EM. In this study, mitochondrial abnormalities were defined as the presence of at least one of the following features: COX-negative fibers, ragged-red fibers (RRF), or ragged-blue fibers (RBF).

Histopathological features were further analyzed for the patients who had a biopsy in our center and also tested for anti-cN1A antibody. The degree of inflammatory cells was scored according to Pinto et al. on a scale from 0 (absence), 1 (minimal scattered inflammation), 2 (1 small collection per 5×field), 3 (2 small collections per 5×field), to 4 (3 or more small collections per 5×field) (21). C5b-9 staining was scored according to Yang et al. on a scale from 0 (absence), 1 (<3% of muscle fibers in whole biopsy), 2 (3–10% of muscle fibers in whole biopsy), 3 (11–30% of muscle fibers in whole biopsy), to 4 (>30% of muscle fibers in whole biopsy) (22). MHC-I staining was scored according to Lindvall et al. on a scale from 0 (negative on muscle fibers), 1 (expression on parts of the muscle fiber membrane in parts of the muscle biopsy), 2 (expression on parts of the muscle fiber membrane throughout the biopsy), 3 (expression involving the total of the muscle fiber membrane), 4 (3 with addition groups of dark, granulated muscle fibers) to 5 (3 with granulated muscle fibers present throughout the biopsy) (23). The amount of COX-negative fibers and p62 deposition were scored semi-quantitatively according to Pinto et al. on a scale from 0 (absence), 1 (≤3 of muscle fibers in whole biopsy), 2 (>3 of muscle fibers in the whole biopsy, and 0–1 muscle fiber in a 10× field), 3 (1–2 of muscle fibers in a 10×field), to 4 (≥2 of muscle fibers in a 10×field) (21).

### Statistical analysis

Continuous variables that followed a normal distribution were summarized as mean ± standard deviation, and continuous variables that were not normally distributed were presented as median (interquartile range, IQR). Categorical variables were summarized as counts (percentages). The cohort was stratified according to several clinically relevant aspects, including sex, age at onset, dysphagia, anti-cN1A antibody status, and complement deposition. Age at onset was categorized as <50 years group and ≥50 years group. Complement deposition was classified as score >2 group and score ≤2 group. Subgroup comparisons were assessed by using the independent samples t-test or the Mann-Whitney U test for continuous variables, and the Chi-square test for categorical variables.

Further validation was performed for outcomes in unadjusted analyses by using analysis of covariance (ANCOVA) for continuous outcomes and binary logistic regression for categorical outcomes. Adjustment analyses were performed for predefined relevant covariates, including sex, age at onset, disease duration and age at biopsy. A p-value of less than 0.05 was considered statistically significant. All statistical analyses were performed using IBM SPSS Statistics 27.

## Results

### Demographics and clinical findings

**Figure 1:**
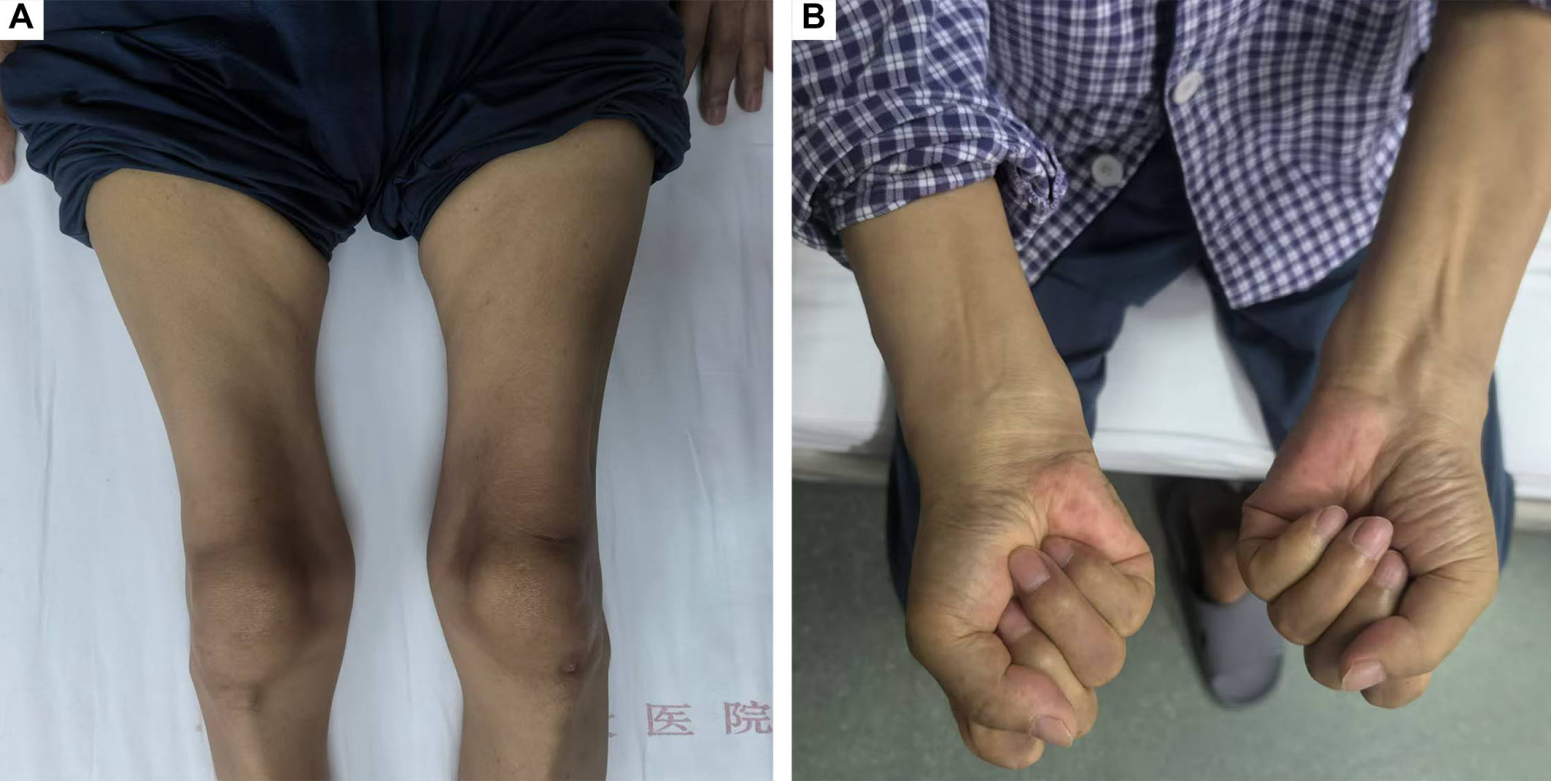
Typical distribution of muscle atrophy in a patient with IBM. **(A)** Muscle atrophy in the bilateral quadriceps muscles. **(B)** Muscle atrophy in the bilateral forearms and weakness in hand grip and finger flexion.

This retrospective study identified 43 IBM patients fulfilling the 2024 ENMC criteria between January 2004 and May 2024. Thirty-six patients performed muscle biopsy in our center. The total number of muscle biopsies for patients aged over 18 years old was 6,535 during this period, and the proportion of IBM was 0.55% (36/6,535) in our center. (**Table 1**). Twenty-five (58.1%) were males, with a male-to-female ratio of 1.4:1. The mean age at onset was 54.9 ± 9.6 years (ranged from 30 to 78 years). Five patients (11.6%) presented with their first symptoms before the age of 45. The median diagnostic interval was 5 years (IQR 3 to 6 years). The most common symptom at onset was weakness in bilateral proximal lower limbs (n = 32, 74.4%). Three patients (7.0%) presented with weakness in both upper and lower limbs, and two patients (4.7%) presented with proximal upper limb weakness. Two patients (4.7%) presented with dysphagia as the first symptom. At the time of clinical assessment, all the patients developed weakness in hip flexion, 38 patients (88.4%) presented weakness in knee extension, and 40 patients (93.0%) presented with weakness in finger flexion. (**Table 1**). Notably, the strength of knee extension was weaker than hip flexion in the majority of the patients (n = 29, 67.4%). Thirty-five patients (81.4%) presented with the typical clinical features of weakness in both knee extension and finger flexion, and muscular atrophy in the bilateral thighs and forearms was shown in **Figure 1**. In addition, 30 patients (69.8%) developed weakness in neck flexion, and 17 patients (39.5%) developed dysphagia. (**Table 1** and **Supplementary Table 2**).

**Table 1:** Clinical characteristics of the overall cohort and comparisons by sex.

Characteristics	Total	Sex group		
		Female	Male	P
Demographics, n=43				
Female (%)	18/43(41.9)	—	—	—
Age at onset, yrs	54.9 ± 9.6	54.4 ± 10.7	55.2 ± 8.9	0.767
Disease Duration, yrs	5.0 (3.0,6.0)	4.5(3,7)	5(3,7)	0.765
Age at biopsy, yrs	60.1 ± 9.5	60.2 ± 10.5	60.1 ± 9.0	>0.999
Clinical feature, n =43				
KE weakness (%)	38/43(88.4)	17/18(94.4)	21/25(84)	0.292
HF weakness (%)	43/43(100)	18/18(100)	25/25(100)	—
KE ≤ HF (%)	33/43(76.7)	12/18(66.7)	21/25(84)	0.275
FF weakness (%)	40/43(93.0)	18/18(100)	22/25(88)	0.128
NF weakness (%)	30/43(69.8)	18/18(100)	12/25(48)	<0.001^*^
Strength sum scores	99.6 (87.5,108.5)	97.05 (85.675,105.675)	102.5 (87.75,109.4)	0.362
Upper limb muscle strength	59.0(52.0,64.9)	55.8(51.45,65.275)	61.9(53.164.8)	0.284
Lower limb muscle strength	42.0(36.0,45.0)	41.75(36,44)	42.5(34.25,46.25)	0.579
Dysphagia (%)	17/43(39.5)	12/18(66.7)	5/25(20)	0.002^**^
IBMFRS scores	33(29,37)	32.5(28.75,35.5)	33(29,37.5)	0.666
CK, IU/L	865(401,1200)	780.5(338.25,1345)	908(405.5,1403)	0.530
Muscle biopsy feature				
Endomysial inflammation (%), n=43	43/43(100)	18/18(100)	25/25(100)	—
Perimysial inflammation (%), n =43	11/43(25.6)	7/18(38.9)	4/25(16)	0.090
Invasion or surrounding of non-necrotic muscle fibers (%), n=43	38/43(88.4)	16/18(88.9)	22/25(88)	0.929
RV (%), n=43	34/43(79.1)	11/18(61.1)	23/25(92)	0.014^***^
MHC-I positivity (%), n=36	36/36(100)	15/15(100)	21/21(100)	—
Mitochondrial abnormalities (%), n=39	30/39(76.9)	13/15(86.7)	17/24(70.8)	0.437
Endomysial fibrosis (%), n=37	28/37(75.7)	12/15(80)	16/22(72.7)	0.711
Complement positive (%), n=31	24/31(77.4)	9/11(81.8)	15/20(75)	>0.999
p62 deposition (%), n=27	25/27(92.3)	9/10(90)	16/17(94.1)	0.760

Data were presented as median (IQR) or n/N (%) (N represented data available for analysis per variable); yrs = years; KE, knee extension; HF, hip flexion; FF, finger flexion; NF, neck flexion; IBMFRS, inclusion body myositis functional rating scale; CK, creatine kinase; RV, rimmed vacuoles; MHC, major histocompatibility.

^*^Only unadjusted analysis was performed due to complete separation arising from a 100% event rate in this subgroup.

^**^Adjusted for disease duration and age at onset, P = 0.005, OR = 8.57, 95% CI 1.95 to 37.73.

^***^Adjusted for age at onset and age at biopsy, P = 0.027, OR = 0.14, 95% CI 0.02 to 0.79.

Bold values indicate statistically significant results with a P-value < 0.05.

IBMFRS was evaluated in 39 patients with a median score of 33 (IQR 29–37). Serum CK levels were within normal range (≤170 IU/L) in 24 patients (55.8%). Two patients (4.7%) showed a marked elevation of CK level over 15 times of the upper limit of normal (>2550 IU/L). The overall median CK level was 865 IU/L (range from 38 to 4000). As for comorbidities, seven patients (16.3%) had Sjögren’s syndrome (SS), four patients (7.0%) had possible SS or undifferentiated connective tissue disease, 12 patients (27.9%) had coronary heart disease, 18 patients (41.9%) had hypertension, 12 patients (27.9%) had diabetes mellitus, one patient (2.3%) had chronic hepatitis B virus (HBV) infection, one patient (2.3%) had chronic hepatitis C virus (HCV) infection, and one patient (2.3%) had monoclonal gammopathy of undetermined significance (MGUS). (**Table 1** and **Supplementary Table 2**).

None of the patients reported a family history of neuromuscular diseases or genetic disorder. Whole-exome sequencing (WES) was performed in 23 patients, and a variant in the *SQSTM1* gene was identified in one patient (unpublished data).

### Muscle biopsy findings

**Figure 2:**
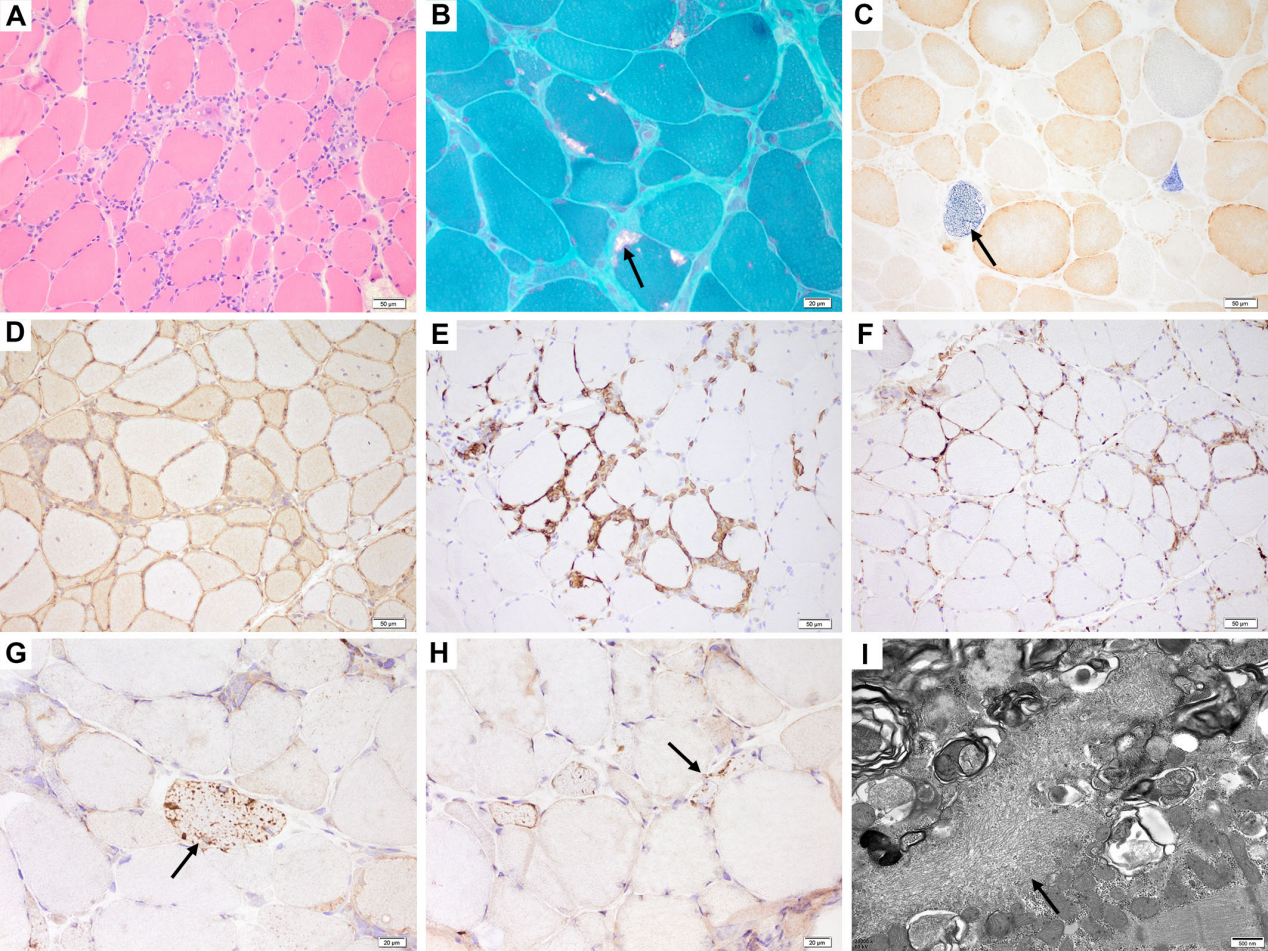
Muscle biopsy in vastus lateralis of a patient with IBM. **(A)** H&E staining showed endomysial inflammatory cells invasion and surrounding non-necrotic muscle fibers. **(B)** mGT staining confirmed the presence of RVs (arrow). **(C)** COX/SDH staining showed multiple COX-negative fibers (arrow). **(D)** Diffuse MHC-I up-regulation in the sarcolemma and cytoplasm of muscle fibers. **(E)** CD8 immunohistochemistry revealed cytotoxic T-cells infiltration. **(F)** C5b-9 staining showed complement deposition on the sarcolemma of non-necrotic fibers. **(G)** p62 protein aggregates within fibers (arrow). **(H)** TDP-43 mislocalization to the cytoplasmic of muscle fibers (arrow). **(I)** The tubulofilamentous inclusions were confirmed by EM (arrow). Scale bars were 50 μm in **(A, C-F)**, 20 μm in **(B, G, H)**, and 500 nm in **(I)** .

All the patients had a muscle biopsy, mostly in the quadriceps femoris (n = 22, 51.2%) and biceps brachii (n = 19, 44.2%), and only one in the tibialis anterior and gastrocnemius muscles, respectively. Thirty-eight patients were performed muscle biopsies in our center. (**Table 1** and **Supplementary Table 2**). Endomysial inflammatory infiltrates were observed in all the patients, and lymphocytes invasion or surrounding of non-necrotic muscle fibers was seen in 38 (88.4%) of them. (**Figure 2A**). Rimmed vacuoles and mitochondrial abnormalities were found in 34 patients (79.1%) and 30 patients (76.9%), respectively. (**Figures 2B, C**). Immunohistochemistry stainings showed up-regulation of MHC-I expression in all the patients, and CD8-positive T-cell infiltration in 30 patients (93.8%). (**Figures 2D, E**) Complement deposition on the sarcolemma of non-necrotic muscle fibers was observed in 24 patients (77.4%). (**Figure 2F).** In addition, perimysitis and perivascular inflammation were also observed in 11 patients (25.6%) and 15 patients (34.9%), respectively. P62 staining was performed in 27 patients, and p62 protein aggregations were observed in 25 of them (92.6%). (**Figure 2G**). TDP-43 staining was performed in 14 patients, and TDP-43 mislocalization was observed in 12 patients (85.7%). (**Figure 2H**). EM was performed in 23 patients, and tubulofilamentous inclusions were found in 15 patients (65.2%). (**Figure 2I**).

### Correlation analysis

Comparisons between age-at-onset groups showed no significant differences in either clinical or pathological features. (**Supplementary Table 3**). The differences between sex were also analyzed. (**Table 1**). After adjustment for disease duration and age at onset, dysphagia was more frequent in females than the males (P = 0.005). Neck flexor weakness was present in all the females compared with 48% of males, with statistically significant difference (P < 0.001). Histopathological findings showed that female patients had a significantly lower frequency of RVs in muscle biopsies (P = 0.027).

Patients were stratified by the presence or absence of dysphagia for comparative analysis. (**Table 2** and **Supplementary Table 4**). The initial unadjusted analysis indicated that dysphagia was associated with weakness in neck flexion and upper limb muscle strength, particularly in wrist flexion. However, these differences were no longer statistically significant after adjustment for age at onset, disease duration, and sex.

**Table 2:** Comparison of clinical and pathological characteristics between the subgroups of dysphagia.

Characteristics	with dysphagia	without dysphagia	P
Demographics, n = 43			
Female (%)	12/17(70.6)	6/26(23.1)	0.002^*^
Age at onset, yrs	52.9 ± 8.8	56.1 ± 10.0	0.390
Disease Duration, yrs	5(3,10)	4(3,5.25)	0.196
Age at biopsy, yrs	59.4 ± 8.4	60.6 ± 10.4	0.718
Clinical feature, n = 43			
KE weakness (%)	16/17(94.1)	22/26(84.6)	0.342
HF weakness (%)	17/17(100)	26/26(100)	—
KE ≤ HF (%)	12/17(70.6)	21/26(80.8)	0.440
FF weakness (%)	16/17(94.1)	24/26(92.3)	0.820
NF weakness (%)	16/17(94.1)	14/26(53.8)	0.005^**^
Strength sum scores	92.6(83.5,104.4)	102.95(91,109.85)	0.112
Upper limb muscle strength	54.2(50.45,61.15)	61.95(55.75,66)	0.047^***^
Lower limb muscle strength	41.5(34.5,44)	42.5(36,45.25)	0.551
IBMFRS scores	32(29,36.5)	33.5(29.75,38.25)	0.575
CK, IU/L	860(38,3538)	920.5(399,1301.5)	0.990
Muscle biopsy feature			
Endomysial inflammation (%), n=43	17/17(100)	26/26(100)	—
Perimysial inflammation (%), n =43	6/17(35.3)	5/26(19.2)	0.238
Invasion or surrounding of non-necrotic muscle fibers (%), n=43	14/17(82.4)	24/26(92.3)	0.319
RV (%), n=43	12/17(70.6)	22/26(84.6)	0.269
MHC-I positivity (%), n=36	16/16(100)	20/20(100)	—
Mitochondrial abnormalities (%), n=39	12/15(80)	18/24(75)	>0.999
Endomysial fibrosis (%), n=37	11/15(73.3)	17/22(77.3)	>0.999
Complement positive (%), n=31	10/13(76.9)	14/18(77.8)	>0.999
P62 deposition (%), n=27	7/8(87.5)	18/19(94.7)	0.232

Data were presented as median (IQR) or n/N (%) (N represented data available for analysis per variable); yrs, years; KE, knee extension; HF, hip flexion; FF, finger flexion; NF, neck flexion; IBMFRS, inclusion body myositis functional rating scale; CK, creatine kinase; RV, rimmed vacuoles; MHC, major histocompatibility.

*Adjusted for disease duration and age at onset, P = 0.004, OR = 8.35, 95% CI 1.94 to 35.95.

**Adjusted for disease duration, age at onset, P = 0.023, OR = 16.08, 95%CI 1.46 to 176.93. Additionally adjusted for sex, P = 0.171, OR = 5.49, 95% CI 0.48 to 62.80.

***Adjusted for disease duration, age at onset, and sex, P = 0.289, adjusted mean difference = -3.67, 95%CI -10.59 to 3.24, partial η^2^ = 0.03.

Bold values indicate statistically significant results with a P-value < 0.05.

### Anti-cN1A antibodies in IBM

Thirty patients were tested for anti-cN1A antibodies. (**Table 3**). Twenty patients (66.7%) were seropositive and 10 (33.3%) were seronegative. Specifically, the positivity of anti-cN1A antibody was detected in 7/14 (50.0%) patients by ELISA and 13/16 (81.3%) patients by CBA. After adjustment for disease duration and age at onset, the seropositive group was associated with a later age at onset (P = 0.034). Histopathological analyses showed that the seropositive group had a higher score for COX-negative fibers (≥ 3) compared with the seronegative group (P = 0.039). However, this difference was no longer significant after adjustment for age at biopsy.

**Table 3:** Comparison of clinical and pathological characteristics between the subgroups of Anti-cN1A antibody^a^.

Characteristics	cN1A-negative	cN1A-positive	P
Demographics, n = 30			
Female (%)	6/10(60)	7/20(35)	0.255
Age at onset, yrs	49 ± 8.4	57.3 ± 9.6	0.019^*^
Disease duration, yrs	8(1,12)	4.5(1,6)	0.155
Age at biopsy, yrs	55.8 ± 9.1	61.5 ± 10.2	0.143
Clinical feature, n =30			
KE weakness (%)	9/10(90)	17/20(85)	>0.999
HF weakness (%)	10/10(100)	20/20(100)	—
KE ≤ HF (%)	7/10(70)	15/20(75)	>0.999
FF weakness (%)	10/10(100)	19/20(95)	>0.999
NF weakness (%)	7/10(70)	13/20(65)	>0.999
Strength sum scores	101.25(70.7,109.8)	102.4(82.4,117.6)	0.307
Upper limb muscle strength	59.875(33.2,65.5)	61.1(44.4,70)	0.448
Lower limb muscle strength	42(25,47)	42.25(33.5,48.5)	0.619
IBMFRS scores	34.5(29,42)	34(25,43)	0.812
Dysphagia (%)	5/10(50)	6/20(30)	0.425
CK, IU/L	932.5(38,2180)	559.5(106,4000)	0.448
Muscle biopsy feature			
Endomysial inflammation (%), n=30	10/10(100)	20/20(100)	—
Perimysial inflammation (%), n=30	2/10(20)	7/20(35)	0.675
Invasion or surrounding of non-necrotic muscle fibers (%), n=30	9/10(90)	18/20(90)	>0.999
Endomysial fibrosis (%), n=24	7/8(87.5)	11/16(68.8)	0.621
RV (%), n=30	7/10(70)	18/20(90)	0.300
MHC-I positivity (%), n =28	9/9(100)	19/19(100)	—
Mitochondrial abnormalities (%), n=26	7/9(77.8)	17/17(100)	0.111
COX-negative fibers score ≥ 3(%), n = 23	2/8(25)	11/15(73.3)	0.039^**^
P62 deposits score ≥ 4(%), n = 22^b^	2/8(25)	10/14(71.4)	0.074
Deposition of the complement score > 2(%), n =23	1/8(12.5)	7/15(46.7)	0.176

Data were presented as median (IQR) or n/N (%) (N represented data available for analysis per variable); yrs, years; KE, knee extension; HF, hip flexion; FF, finger flexion; NF, neck flexion; IBMFRS, inclusion body myositis functional rating scale; CK, creatine kinase; RV, rimmed vacuoles; MHC, major histocompatibility; COX, cytochromec oxidase.

^a^The positivity of anti-cN1A antibody was detected in 7/14 (50.0%) patients by ELISA and 13/16 (81.3%) patients by CBA.

^b^P62 immunostaining data were available in 22 patients. One case was excluded because the corresponding histological slides were no longer available for reassessment.

*Adjusted for sex and disease duration, P = 0.034, mean difference = -9.82, 95%CI -18.82 to -0.82, partial η^2^ = 0.162.

**Adjusted for sex, age at onset P = 0.036, OR = 0.08, 95%CI 0.01 to 0.85. Adjusted additionally for age at biopsy, P = 0.070, OR = 0.09, 95%CI 0.01 to 1.21.

Bold values indicate statistically significant results with a P-value < 0.05.

### Complement deposition in IBM

The relationship between complement deposition and muscle strength were analyzed in 22 patients (**Table 4** and **Supplementary Table 6**). The patients with higher complement deposition score (>2) were tended to show lower overall muscle strength (P = 0.050). After adjustment for sex, age at onset and disease duration, there was a statistically significant association between complement deposition and more severe decline in muscle strength in proximal upper limbs and distal lower limbs (P = 0.008 and P = 0.006). However, no significant differences were observed in other clinical or pathological features.

**Table 4:** Comparison of clinical and pathological characteristics between the subgroups of complement deposition.

Characteristics	Complement deposits score >2	Complement deposits score ≤2	P
Demographics, n = 23			
Female (%)	3/8(37.5)	7/15(46.7)	>0.999
Age at onset, yrs	56.4 ± 7.5	52.5 ± 12.0	0.414
Disease Duration, yrs	3.5(2.25,5)	5(3,10)	0.650
Age at biopsy, yrs	60.3 ± 7.9	58.1 ± 12.0	0.728
Clinical feature, n = 23			
KE weakness (%)	7/8(87.5)	13/15(86.7)	>0.999
HF weakness (%)	8/8(100)	15/15(100)	—
KE ≤ HF (%)	6/8(75)	12/15(80)	>0.999
NF weakness (%)	4/8(50)	11/15(73.3)	0.371
Ankle dorsiflexion weakness (%)	5/8(62.5)	1/15(6.7)	0.009^*^
Elbow flexion weakness (%)	7/8(87.5)	6/15(40)	0.074
Strength sum scores	90.05(84.175,102.45)	102.5(97.10,110)	0.050
Proximal upper limb muscle strength	23.5(21.75,26.25)	27(25,29)	0.018^**^
Upper limb muscle strength	53.85(45.975,61.125)	61.25(55.6,66)	0.058
Distal lower limb muscle strength	19.5(14.25,20)	20(20,20)	0.026^***^
Lower limb muscle strength	39.75(34.75,42.375)	42.5(40,45)	0.077
IBMFRS scores	34(33,36.75)	34(30,39)	0.334
Dysphagia (%)	2/8(25)	6/15(40)	0.657
CK, IU/L	686(330,1585)	701(334,1937)	0.513
Muscle biopsy feature			
Endomysial inflammation (%), n = 23	8/8(100)	15/15(100)	—
Perimysial inflammation (%), n = 23	4/8(50)	3/15(20)	0.182
Invasion or surrounding of non-necrotic muscle fibers (%), n = 23	7/8(87.5)	13/15(86.7)	>0.999
Endomysial fibrosis (%), n = 23	5/8(62.5)	13/15(86.7)	0.297
RV (%), n = 23	8/8(100)	11/15(73.3)	0.257
MHC-I positivity (%), n = 23	8/8(100)	15/15(100)	—
Mitochondrial abnormalities (%), n = 23	7/8(87.5)	14/15(93.3)	>0.999
COX-negative fibers score ≥3 (%), n = 23	6/8(75)	7/15(46.7)	0.379
p62 deposits score ≥ 4 (%), n =22^a^	4/8(50)	8/14(57.1)	>0.999

Data were presented as median (IQR) or n/N (%) (N represented data available for analysis per variable); yrs, years; KE, knee extension; HF, hip flexion; FF, finger flexion; NF, neck flexion; IBMFRS, inclusion body myositis functional rating scale; CK, creatine kinase; RV, rimmed vacuoles; MHC, major histocompatibility; COX, cytochromec oxidase.

^a^p62 immunostaining data were available in 22 patients. One case was excluded because the corresponding histological slides were no longer available for reassessment.

*Unadjusted comparison only. Logistic regression was not feasible because of complete separation caused by sparse events in this subgroup.

**Adjusted for sex, age at onset and disease duration, P = 0.008, mean difference = -3.77, 95%CI -6.44 to -1.10, partial η² = 0.356.

***Adjusted for sex, age at onset and disease duration, P = 0.006, mean difference = -2.53, 95%CI -4.23 to -0.84, partial η^2^ = 0.342.

Bold values indicate statistically significant results with a P-value < 0.05.

## Discussion

This study characterized the clinical, pathological, and laboratory features of Chinese patients with IBM through a single-center retrospective cohort analysis. The proportion of patients who underwent muscle biopsy who were subsequently diagnosed with IBM in our center was 0.55% (36/6,535). This is lower than a previous report of 0.68% in another Chinese cohort (16). This confirms that IBM is a rare disease in the Chinese population. Due to the rarity of IBM, the sample size of most single-center studies was around 20 to 60 cases (14–16, 24), while only a few specialized centers reported larger cohorts with over 100 patients (20, 25). Demographic data revealed some differences compared with other populations. In this cohort, the male-to-female ratio was 1.4:1, slightly lower than the ratios in Caucasian cohorts (ranging from approximately 1.7:1 to 2.3:1) (5, 20, 26). The mean age at onset of our patients was 54.9 years, while the median or mean age at onset from previous studies was 58.7 to 68.8 years in Caucasian populations (5, 20, 26), and 62.4 to 64.6 years in Japanese cohorts (27, 28). The relatively earlier onset age in this cohort might be due to the inclusion of patients with onset age below 45 years according the revised 2024 ENMC criteria (2). Although a Korean cohort reported a comparable mean age at onset of 54 years (24), their finding might be limited by the small sample size. In our cohort, five patients (11.6%) in our cohort presented with first symptoms before age 45, which suggested the underestimation of early-onset cases. The overall mean age at onset in IBM might be earlier than that was currently reported. Recent studies demonstrated that patients with early onset were associated with more severe disease progression and greater mitochondrial abnormality burden (29, 30). This highlights the importance of early diagnosis and intervention to improve outcomes.

This study confirmed that Chinese IBM patients presented with the characteristically selective muscle weakness pattern, consistent with the reports from Caucasian, Japanese and Korean studies (5, 24, 26–28). The typical features of muscle weakness predominantly in knee extension and finger flexion were observed in the majority of this cohort, confirming this pattern as a core clinical feature of IBM among different populations. Interestingly, all patients in this cohort had weakness in hip flexion, and 88.4% of the patients with weakness in knee extension, while weakness in knee extension was worse than hip flexion in 76.6% of the patients. This indicated that quadriceps were more severely affected while iliopsoas were more frequently affected. This has not been described in previous studies (5, 24, 26–28). Additionally, neck flexion weakness was presented in 69.8% of patients in this cohort, which indicated the involvement of neck flexors might be a common feature in IBM and required careful examination at routine clinical practice.

Dysphagia has been reported to occur in 30% to 80% of IBM patients in previous studies, with higher prevalence associated with longer disease duration (12, 28, 29, 31, 32). In this cohort, 39.5% of patients developed dysphagia during the disease course, while only 4.7% reported it as a symptom at onset. This proportion of initial dysphagia presentation appears lower than that documented in prior reports (28, 31). Unadjusted analysis revealed that patients with dysphagia had weaker upper limb strength, particularly in wrist flexion. However, the statistical significance disappeared after adjustment for disease duration, age at onset, and sex. These findings suggested that dysphagia might reflect overall disease severity rather than representing an isolated symptom. Furthermore, multiple studies have found a direct link between dysphagia and aspiration pneumonia (32–34), which is also a leading cause of death in IBM (35). Therefore, early identifying dysphagia, especially when co-occurring with features like neck flexion weakness, is crucial for the early recognition of high-risk patients, and to take further intervention for complications and improving prognosis.

In this cohort, female patients were more prone to develop dysphagia, which is consistent with previous reports (5, 28). In addition, the female patients had a significantly higher risk of developing weakness in neck flexion than the males (P < 0.001). There was a significant association between dysphagia and neck flexion weakness (P = 0.005), however, the association was no longer significant after adjustment for sex (P = 0.171). This indicated that sex primarily influenced both symptoms. Previous studies suggested that estrogen deficiency in postmenopausal women could lead to neuromuscular impairment in cervical regions (36–38). Given the high prevalence of these symptoms in the female patients, we hypothesize that similar hormonal mechanisms may play a role in IBM, which potentially exacerbates muscle vulnerability in craniocervical regions. The exact pathophysiological link between dysphagia and neck muscle weakness in IBM requires further elucidation. Therefore, clinical management priorities should differ by sex, particularly monitoring dysphagia and neck flexion weakness in the female patients. Regarding the distribution of muscle weakness, our findings partially support previous conclusions. One study indicated that female patients have stronger finger flexor strength than males (20), and a similar trend was observed in our cohort, although it did not reach statistical significance. A recent study suggested that males are more likely to have quadriceps weakness (5), while this was not observed in our study.

The anti-cN1A antibody demonstrates relatively high specificity for IBM, but its diagnostic relevance remains controversial. This study found that seropositive patients had a significantly later age at onset (mean onset age of 57.3 years) after adjustment (P = 0.034). A similar finding has been reported by Felice et al. (14). No significant association was observed between antibody and sex, which was consistent with previous studies (7, 10, 11, 39). Several studies suggested a higher frequency of dysphagia in seropositive patients (11, 12, 40), and some reported association with facial muscle involvement (7). The patterns of muscle weakness associated with seropositive patients varied across different cohorts, including proximal upper limb weakness (7), finger flexion weakness (10, 28), or lower limb involvement (40). While in this cohort, no significant correlations were found between anti-cN1A antibody and specific patterns of muscle weakness. The prognostic significance of anti-cN1A antibodies also remains debated, with conflicting reports regarding survival (7, 10).

In muscle pathology, previous studies have reported a higher frequency of COX-deficient fibers in seropositive patients (7, 10). In our cohort, a similar trend was observed, but it did not reach statistical significance after adjustment for age at biopsy (P = 0.070). These findings suggested that both anti-cN1A antibody and age might play a role in COX deficiency in IBM. There might be differences in pathogenic mechanisms between anti-cN1A subgroups. Paul et al. further indicated that seropositive patients exhibited more frequent auto-aggressive inflammation on muscle biopsy, though not as an isolated pathological feature (13). However, our study found no statistically significant association between antibody status and focal invasion of inflammatory cells or MHC expression, which was consistent with most reports (10, 13, 14, 28). Additionally, high levels of complement deposition (score>2) were observed almost exclusively in seropositive patients. Although this finding did not reach statistical significance, it may provide a clue to the interaction between antibody and complement activation.

Although complement deposition has been reported in isolated IBM cases (41, 42), it has seldom been considered a hallmark pathological feature of IBM (43). Recently, Syed et al. reported the C5b-9 upregulation in 17 of 20 IBM patients (44, 45). Our study further analyzed complement deposition in a defined Chinese IBM cohort. Complement deposition was observed in 77.4% of cases. Patients with higher levels of complement deposition developed more severe muscle weakness with reduced overall muscle strength, especially in elbow flexion and ankle dorsiflexion. These findings highlighted a potential association between complement deposition and disease severity in IBM. Although the complement terminal complex C5b-9 can cause membrane injury, IBM is primarily considered a T-cell mediated and degenerative process (2). Consequently, the complement deposition is likely a secondary phenomenon associated with muscle fiber injury rather than a primary driver. No complement targeting therapies have demonstrated clinical efficacy in IBM to date (44). Therefore, the potential role of complement in pathophysiology of IBM requires further investigation.

Several limitations of this study should be acknowledged. First, as a single-center retrospective cohort analysis, the study carries a risk of selection bias. Second, although this represents the largest IBM cohort in China so far, the limited sample size may have reduced statistical power. In addition, correlation findings should also be interpreted with caution due to the small sample size. In some subgroup analyses, this limitation resulted in complete separation, preventing valid estimation in multivariable models. Furthermore, due to the retrospective design, some data were missing, and the anti-cN1A antibody was tested by two different methods (ELISA and CBA). Previous studies indicated that while the sensitivity of these two assays might be comparable, CBA demonstrated higher specificity for the diagnosis of IBM (2, 28, 39). These limitations highlight the need for prospective multi-center studies across the nation with larger sample sizes, and a standardized antibody testing method is required in future.

## Conclusion

In conclusion, this single-center retrospective study provides a comprehensive profile of a Chinese IBM cohort on clinical, serological, and myopathological characteristics. Our findings further confirmed the clinical heterogeneity of IBM across different ethnicities. We reported a high frequency of complement deposition in muscle tissue and suggested its involvement in local immune-inflammatory processes and disease severity. Future prospective and multi-center studies with larger sample sizes are necessary to validate these observations and to investigate the disease progression and prognosis of IBM patients in China.

## Data Availability

The raw data supporting the conclusions of this article will be made available by the authors, without undue reservation.
