# Supplementary material for: The clinical, serological and myopathological features of a cohort of Chinese patients with inclusion body myositis: a single center analysis
Source: Front Immunol. 2026 Mar 5;17:1782053. doi: 10.3389/fimmu.2026.1782053 (PMC12999901; doi:10.3389/fimmu.2026.1782053)
Supplement: Supplementary file 1 [file Table1.docx]

**Supplementary Table 1: Manual Muscle Testing Grading Criteria and Conversion to the Kendall Scale**

| MRC Scale | Kendall Scale (Points) | Definition |
| --- | --- | --- |
| 0 | 0 | No movement. |
| 1 | 0 | Flicker of movement only. |
| 2- | 1 | Movement possible with gravity eliminated, but the feasible range of motion is >50% and <100%. |
| 2 | 2 | Can complete full range of motion **when gravity is eliminated**. |
| 2+ | 2 | Active movement against gravity but less than 50% of the feasible range of motion. |
| 3- | 3 | Active movement against gravity but the feasible range of motion is >50% and <100%. |
| 3 | 4 | Can complete full range of motion **against gravity**, but cannot against resistance. |
| 3+ | 5 | Can complete full range of motion against gravity with ability to tolerate minimal resistance at the end of the movement. |
| 4- | 6 | Active movement against resistance identical to MRC 4, but the feasible range of motion is >50% and <100%. |
| 4 | 7 | Active movement against resistance, but strength is **less than normal**. |
| 4+ | 8 | Active movement against resistance identical to MRC 4, but the feasible range of motion is >50% and <100%. |
| 5- | 9 | Active movement against **strong resistance** identical to MRC 5, but the feasible range of motion is >50% and <100%. |
| 5 | 10 | Normal strength. |
